# A gene regulatory network for root hair development

**DOI:** 10.1007/s10265-019-01100-2

**Published:** 2019-03-22

**Authors:** Michitaro Shibata, Keiko Sugimoto

**Affiliations:** RIKEN Center for Sustainable Resource Science, 230-0045 Yokohama, Japan

**Keywords:** Root hair, Cell fate, Cell growth, Transcription factor, Gene regulatory network

## Abstract

Root hairs play important roles for the acquisition of nutrients, microbe interaction and plant anchorage. In addition, root hairs provide an excellent model system to study cell patterning, differentiation and growth. Arabidopsis root hairs have been thoroughly studied to understand how plants regulate cell fate and growth in response to environmental signals. Accumulating evidence suggests that a multi-layered gene regulatory network is the molecular secret to enable the flexible and adequate response to multiple signals. In this review, we describe the key transcriptional regulators controlling cell fate and/or cell growth of root hairs. We also discuss how plants integrate phytohormonal and environmental signals, such as auxin, ethylene and phosphate availability, and modulate the level of these transcriptional regulators to tune root hair development.

## Introduction

**Fig. 1:**
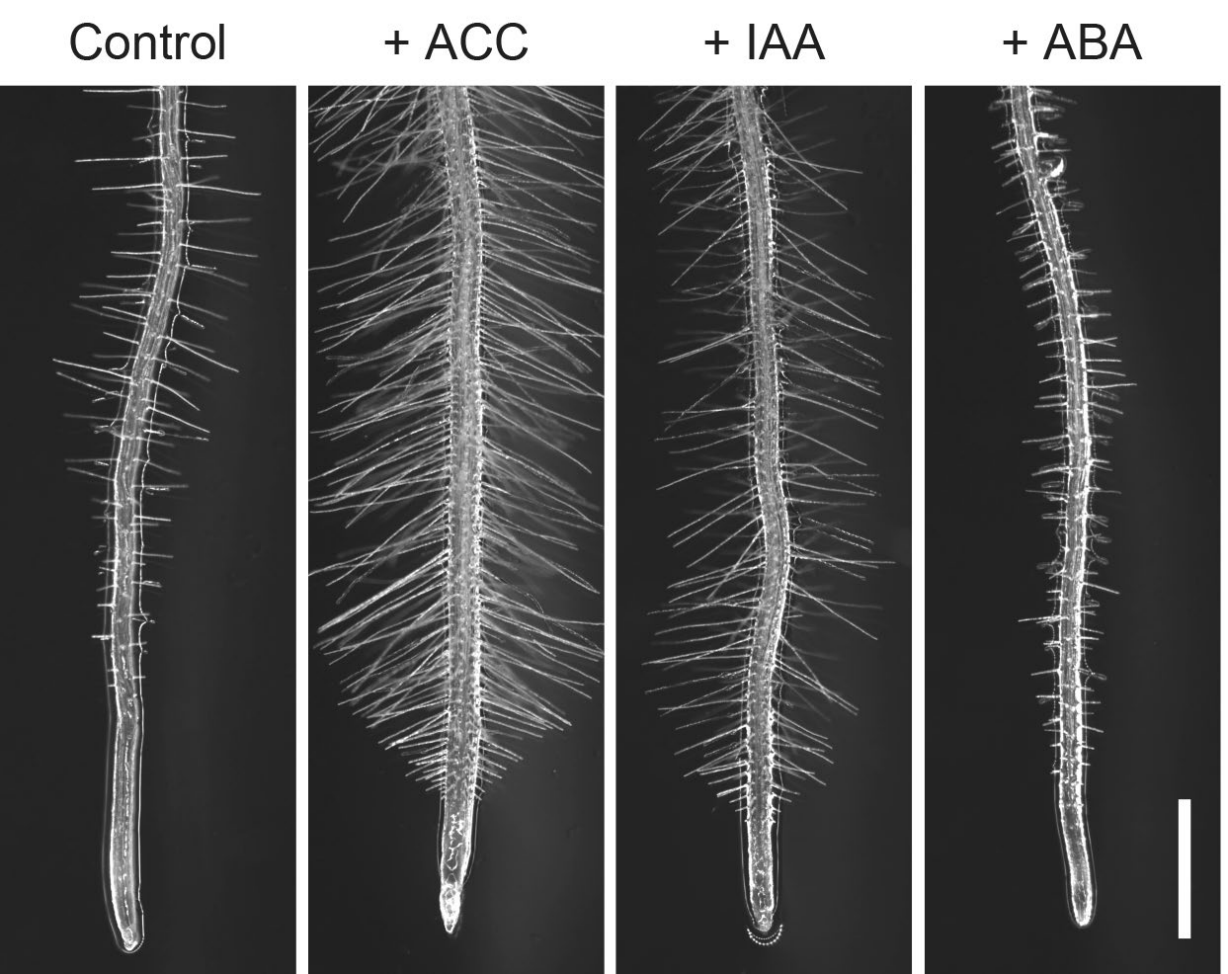
Root hair development is regulated by phytohormones. Arabidopsis seedlings grown on half strength Murashige-Skoog (MS) media with or without phytohormones. Control plants are grown on half strength MS medium without phytohormones. Root hairs are patterned and formed in accordance with the developmental program. Ethylene (100 nM 1-aminocyclopropane-1-1carboxylic acid, ACC) produces ectopic root hairs from non-hair cell files and enhances root hair growth. Auxin (10 nM indole acetic acid, IAA) enhances root hair growth without affecting the cell fate. 50 µM abscisic acid (ABA) inhibits root hair growth. Scale bar = 1 mm

**Fig. 2:**
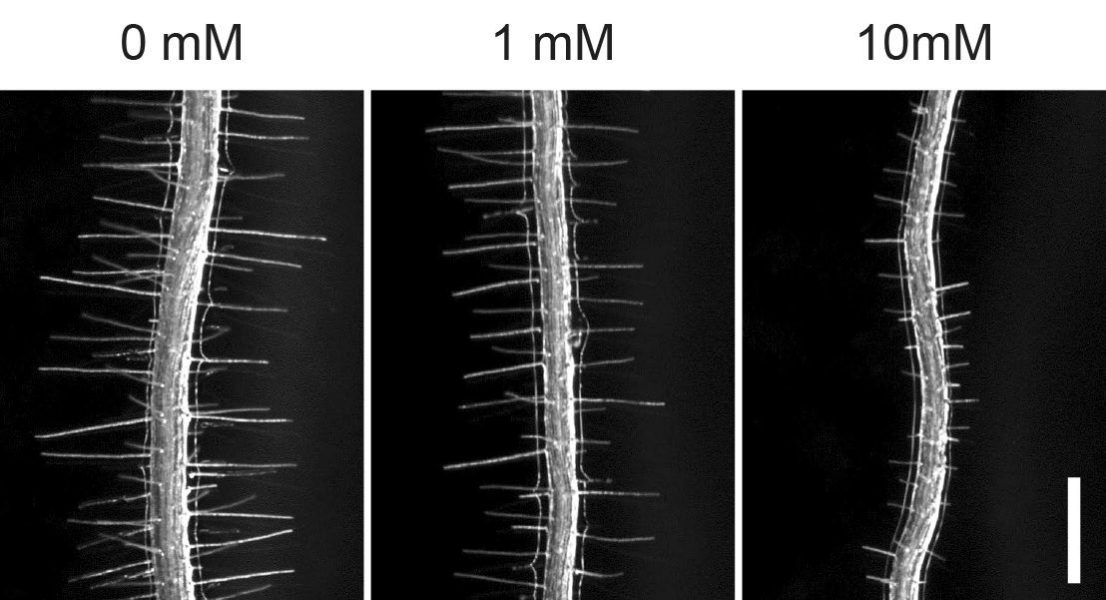
Root hair development is affected by environmental conditions. Phosphate concentration in the media affects root hair development. Scale bar = 500 µm

Root hairs form by tube-like outgrowth of root epidermal cells and they contribute to efficient acquisition of nutrients, microbe interactions and plant anchorage by expanding root surface area. Since root hair development is strongly influenced by surrounding conditions, this regulation is thought to be one of the important mechanisms of environmental adaptation in plants. Indeed, multiple environmental factors, such as availability of phosphorus, nitrogen, iron, magnesium and zinc, as well as phytohormones such as auxin, ethylene and abscisic acid (ABA) alter both cell fate and growth of root hairs (Figs. 1, 2) (Grierson et al. 2014). Root hair growth, for instance, is enhanced under low phosphate conditions but suppressed under high phosphate conditions. Due to these properties, root hairs provide a model system suitable to study cell differentiation and growth in response to environmental signals. In the past decade, various key transcription factors required for optimal root hair formation have been isolated and characterized. Accumulating evidence suggests that a gene regulatory network consisting of these transcription factors has an important role for their dynamic and robust responses. In this review we summarize the transcriptional regulation of root hair development and discuss how plants achieve the optimal response to adapt to environmental stimuli.

## Developmental regulation of root hairs

### Cell fate specification

During early root development, epidermal cells differentiate into either trichoblasts, i.e., root hair-forming cells or atrichoblasts, i.e., non-hair forming cells. In general, plants develop root hairs in one of the following three patterns (Datta et al. 2011). In plants that undergo type I patterning, such as rice, all epidermal cells have the potential to differentiate into root hairs and they develop root hairs in a random manner. For plants that exhibit type II patterning, including Brachypodium, root hair fate is specified by asymmetric cell division, thus smaller daughter cells gain root hair identity while larger daughter cells become non-hair cells. For plants that undergo type III patterning, such as Arabidopsis, hair cell identity is determined by the position of cells. In these plant species, epidermal cells with two underlying cortical cells become hair cells while those with only one underlying cortical cell become non-hair cells, resulting in the formation of hair cell files and non-hair cell files (Datta et al. 2011).

**Fig. 3:**
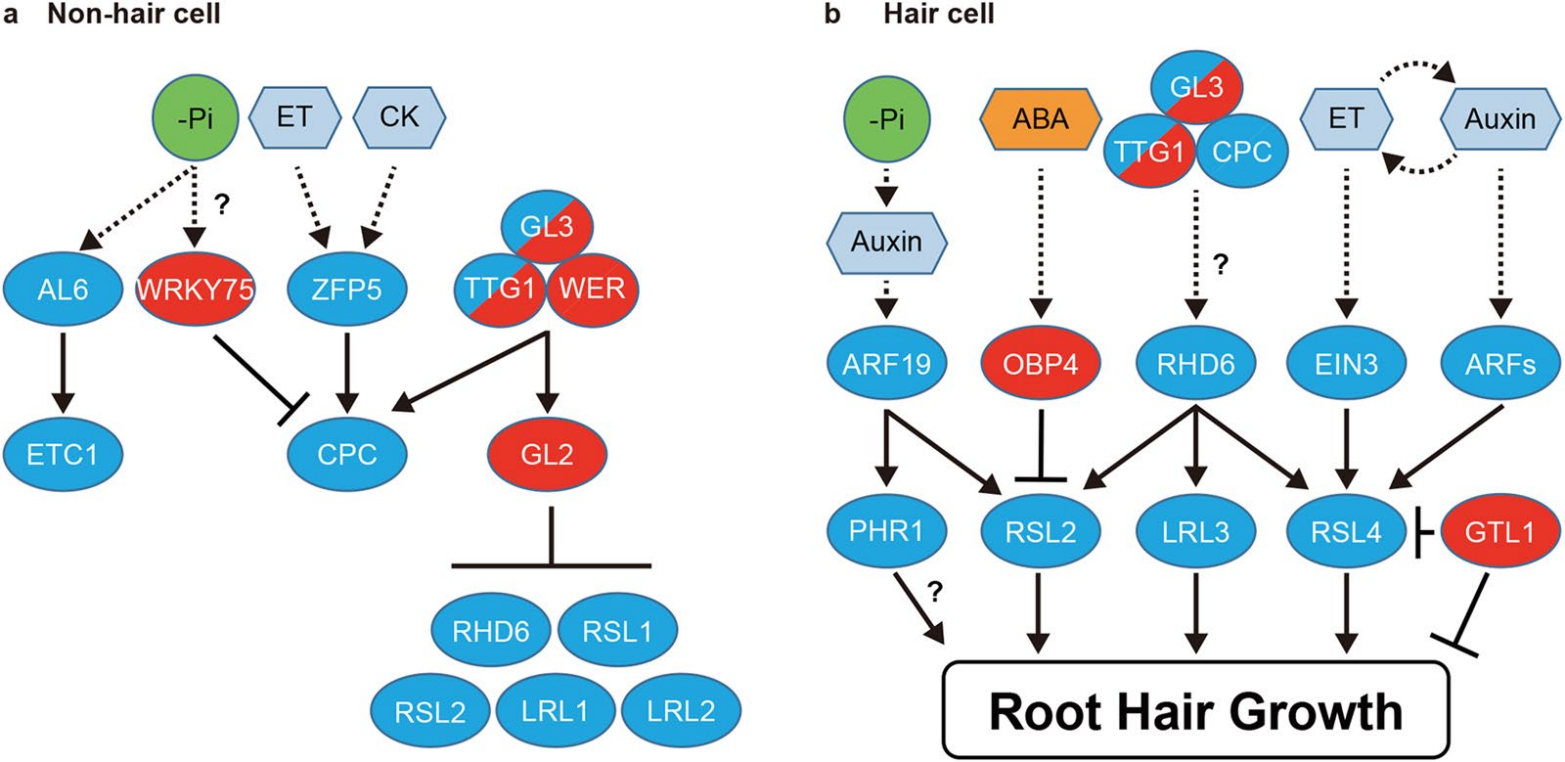
Gene regulatory network to control root hair development. Root hair development is a flexible response to developmental and environmental signals due to the gene network consisting of transcription factors. Exogenous and endogenous signals are transduced by distinct regulators. **a** In non-hair cells, a protein complex consisting of WER, GL3 and TTG1 induces *GL2* expression. GL2 suppresses a set of transcription factors to prevent root hair formation. In addition, the WER-GL3-TTG1 complex induces *CPC* which moves to a hair cell. Although *ZFP5* is dominantly expressed in hair cells and protein mobility is not characterized, ZFP5 is categorized in non-hair cell because ZFP5 directly regulates *CPC*. For the same reason, WRKY75 and AL6 are categorized in non-hair cell. **b** In hair cells, exogenous and endogenous signals are integrated into RSL2, RSL4 and LRL3 to change cell growth. Whether PHR1 regulates root hair growth directly or indirectly is not clear. GTL1 suppresses hair growth both directly and indirectly. Transcriptional activation and repression are indicated by arrows and blunted lines, respectively. The proteins marked in blue and red show positive and negative, respectively, regulators for root hair development. The circles containing blue and red show that they work both positively and negatively for root hair development. For two protein complexes of WER-GL3-TTG1 and CPC-GL3-TTG1, redundant proteins are not shown in the model. Note that protein mobility is not reflected in this model

In the past decade, molecular mechanisms that specify cell fate of root hair have been extensively studied in Arabidopsis. It is now evident that a group of transcription factors have a key role in cell fate determination. In non-hair cells, a R2R3-type MYB transcription factor WERWOLF (WER) (Lee and Schiefelbein 1999), a basic helix-loop-helix (bHLH)-type transcription factor GLABLA3 (GL3) (Bernhardt et al. 2003) or its homolog ENHANCER OF GLABLA3 (EGL3) (Bernhardt et al. 2003), and a WD repeat protein TRANSPARENT TESTA GLABLA1 (TTG1) (Galway et al. 1994) form a protein complex and enhance the expression of *GLABLA2* (*GL2*) which functions as a negative regulator of root hair formation (Masucci et al. 1996; Di Cristina et al. 1996). Lack of GL2 function causes ectopic root hair formation from non-hair cell files. Interestingly, the ectopic root hairs in *gl2* mutants still keep non-hair cell properties such as early vacuolation and longer cell length (Masucci et al. 1996). From this perspective, GL2 seems to promote non-hair cell morphology rather than “cell fate”. To specify root hair identity, a mobile R3-type MYB transcription factor, CAPRICE (CPC) plays a key role (Wada et al. 1997). *CPC* is transcriptionally induced by the WER-GL3-TTG1 complex in non-hair cells and its protein moves to neighboring cells to induce root hairs. In these hair forming cells, CPC forms a protein complex with GL3 (or EGL3) and TTG1. In addition, TRIPTYCON (TRY) (Schellmann et al. 2002) and ENHANCER OF TRY AND CPC 1 (ETC1) (Simon et al. 2007), two homologs of CPC, function in a partially redundant manner with CPC (Bruex et al. 2012; Grierson et al. 2014; Ishida et al. 2008; Salazar-Henao et al. 2016). ETC1, however, does not show cell-to-cell movement like CPC, instead ETC1 moves from sub-epidermal cells to root hair cells (Rishmawi et al. 2018; Tominaga-Wada et al. 2017). ETC1 may have a different role from CPC on root hair development. In any case, CPC and its homologs are essential for the induction of a bHLH transcription factor *ROOT HAIR DEFECTIVE 6* (*RHD6*) which plays key roles in the determination of root hair identity (Fig. 3a) (Masucci and Schiefelbein 1994; Menand et al. 2007).

### Growth of root hair cells

After cell fate specification, the developmental program proceeds cell autonomously and hair-forming cells undergo extensive cell expansion to reach their maximum size. In young root hair cells, RHD6 and its homolog RHD6-LIKE1 (RSL1) promote transcription of genes encoding other bHLH transcription factors RSL2, RSL4 and Lotus japonicus ROOTHAIRLESS-LIKE 3 (LRL3) (Fig. 3a) (Karas et al. 2009; Masucci and Schiefelbein 1994; Menand et al. 2007; Yi et al. 2010). Among these bHLH homologs, RSL4 is a key regulator of root hair growth because constitutively expressed RSL4 proteins, using a *CaMV 35S* promoter, are sufficient to promote root hair growth (Yi et al. 2010). Transcriptome analyses of knock-out, overexpression and inducible lines of *RSL4* identified very few transcription factors as downstream targets of RSL4, suggesting that RSL4 functions at the bottom of a gene regulatory network underling root hair growth (Mangano et al. 2017; Vijayakumar et al. 2016; Yi et al. 2010). Recent molecular genetic studies have demonstrated that RSL4 promotes transcription of *ROOT HAIR SPECIFIC* (*RHS*) genes that are predominantly expressed in root hairs (Hwang et al. 2017; Kim et al. 2006; Won et al. 2009). The promoter sequence of *RHS* genes contains a *cis*-element named Root Hair Element (RHE) and RSL4 binds this sequence to promote their expression in root hairs. Some RHS proteins, as well as other indirect targets of RSL4, are implicated in cell wall biogenesis and remodeling, ROS production or vesicle trafficking (Kim et al. 2006; Won et al. 2009), highlighting the key cellular processes central for root hair growth downstream of RSL4 regulation.

In addition to RSL4, RSL2 promotes root hair growth since the *rsl2* single mutation causes shorter root hairs and the *rsl2 rsl4* double mutation results in complete hairlessness (Mangano et al. 2018; Yi et al. 2010). Unlike RSL4, however, overexpression of *RSL2* does not enhance root hair growth, indicating that RSL2 alone is not sufficient to promote hair growth (Yi et al. 2010). LRL3 also likely promotes root hair growth downstream of RHD6. Although *lrl3* single mutant does not show root hair phenotypes, the *lrl1 lrl3* or *lrl2 lrl3* double mutants develop shorter root hairs (Karas et al. 2009). Unlike LRL3, expression of LRL1 and LRL2 does not require CPC, CPC-like MYBs, RHD6 and RSL1 (Karas et al. 2009). Thus, LRL1 and LRL2 might function in a pathway parallel to the CPC-RHD6 pathway. Further elucidation of LRL downstream targets will be key to work out how these pathways regulate hair growth coordinately.

**Fig. 4:**
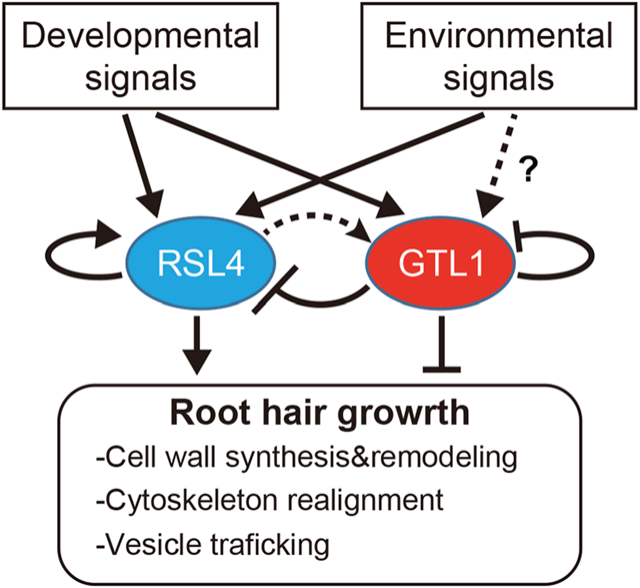
The RSL4-GTL1 module in the gene regulatory network for root hair development. Both environmental signals and developmental signals affect *RSL4* expression. Whether environmental signals affect *GTL1* expression is not clear but *GTL1* is induced by programed developmental signals. RSL4 promotes expression of its own, *GTL1* and genes involved in root hair growth. GTL1 counteracts with RSL4 by suppressing expression of *RSL4*, expression of its own and its downstream targets. This RSL4-GTL1 module might function as a modulator that integrates developmental signals and environmental signals to coordinate root hair growth. Transcriptional activation and repression are indicated by arrows and blunted lines, respectively

Several recent studies have also demonstrated that root hair growth is actively repressed by transcriptional regulation. A trihelix transcription factor GT2-LIKE1 (GTL1) was initially isolated as a repressor of trichome cell growth (Breuer et al. 2009, 2012). Although the single *gtl1* mutant does not show phenotypes in root hairs, the double mutation in *GTL1* and its closest homolog *DF1* prolongs elongation of root hairs (Shibata et al. 2018). Furthermore, root hair specific overexpression of *GTL1* or *DF1*, using an *EXPANSIN A7* promoter, strongly suppresses root hair growth, indicating that both GTL1 and DF1 act as negative regulators of root hair growth (Shibata et al. 2018). Both GTL1 and DF1 bind the *RSL4* promoter region and directly suppress its expression. Interestingly, GTL1, in addition, suppresses expression of many RSL4 target genes as well as its own expression (Shibata et al. 2018). Conversely, RSL4 can induce *GTL1* expression, although probably in an indirect manner (Shibata et al. 2018; Vijayakumar et al. 2016) and it can promote its own expression (Hwang et al. 2017). Therefore, RSL4 and GTL1 form a core transcriptional module to regulate each other and their common downstream targets (Fig. 4). Although the physiological role of this module has not been clarified, the RSL4-GTL1 module may provide robustness and/or flexibility to root hair growth so that they can respond to multiple environmental stimuli adequately (Shibata et al. 2018).

In addition to GTL1 and DF1, a DOF-type transcription factor OBF BINDING PROTEIN 4 (OBP4) is characterized as a negative regulator of root hair growth since induction of *OBP4* inhibits root hair growth (Rymen et al. 2017). Unlike GTL1 and DF1, OBP4 represses *RSL2* expression and does not affect *RSL4* expression, suggesting that plants have at least two transcriptional pathways to repress root hair growth (Fig. 3a). OBP4 is implicated in ABA response (Rymen et al. 2017) and we will discuss further mechanistic details on this in the next section. LRL4 and LRL5 are also reported as a negative regulator of root hair growth since their ectopic overexpression, using a *CaMV 35S* promoter, causes short root hairs (Breuninger et al. 2016; Bruex et al. 2012). It is interesting that LRL4 and LRL5 have negative roles on root hair growth even though other bHLH transcription factors act as positive regulators.

In non-hair cells, GL2 suppresses expression of *RHD6, RSL1, RSL2, LRL1* and *LRL2* to prevent ectopic outgrowth of root hairs (Fig. 3b). Ectopic expression of LRL1 or LRL2 using a *GL2* promoter causes ectopic hair formation, suggesting that LRL1 and LRL2 regulate both root hair growth and cell fate determination (Fig. 3b) (Lin et al. 2015).

## Physiological and environmental modulation of root hair growth

Previous studies have revealed that phytohormones such as auxin, ethylene, cytokinin, strigolactone and jasmonic acid promote root hair growth while ABA and brassinosteroid inhibit root hair growth (Fig. 1) (Grierson et al. 2014; Lee and Cho 2013). Among these phytohormones, auxin and ethylene synergistically regulate root hair growth (Pitts et al. 1998), and a transcriptome analysis uncovered that almost 90% of genes are commonly upregulated by auxin and ethylene (Bruex et al. 2012). In addition, strigolactone-, jasmonic acid- and brassinosteroid-mediated signaling pathways eventually integrate with auxin- and/or ethylene-mediated pathways to coordinate root hair growth (Lee and Cho 2013). Root hair growth is also strongly influenced by nutrient availability in soil and the effect of inorganic phosphate (Pi) is best characterized to date (Fig. 2) (Salazar-Henao et al. 2016). In this section we describe the key direct transcriptional link that translates these hormonal and environmental responses into root hair growth.

### Auxin

Auxin is a well-known phytohormone that massively enhances root hair growth (Fig. 1). Defects in auxin biosynthesis or signaling in mutants such as *tryptophan aminotransferase of arabidopsis 1* (*taa1), transport inhibitor response 1* (*tir1)* and *auxin resistant 1 (axr1-3)* cause hairless or short hair phenotypes, clearly showing that auxin is essential for root hair formation (Velasquez et al. 2016). Auxin does not confer hair cell identity to root epidermal cells because exogenous auxin treatment does not cause ectopic root hair formation. Auxin induces *RSL4* expression, and several activator AUXIN RESPONSE FACTORs, such as ARF5, ARF7, ARF8 and ARF19, bind the *RSL4* promoter to induce its expression (Mangano et al. 2017). A recent study, in addition, demonstrated that auxin also upregulates *RSL2* expression through ARF19 although whether ARF19 directly binds to the promoter sequence of *RSL2* is not yet established (Bhosale et al. 2018). Choi et al. (2018) further reported that root hair specific overexpression of repressor ARFs, i.e., ARF1-4, 9–11 and 16, using an *EXPANSIN7* promoter inhibits root hair growth. These results suggest that auxin can repress root hair growth through these repressive ARFs and this response is transduced locally within root hairs (Choi et al. 2018). Membrane-anchored MYB (maMYB) is also an auxin responsive transcription factor because expression of *maMYB* is elevated by exogenous auxin treatment (Slabaugh et al. 2011). The knock-down mutation of *maMYB* by RNAi reduces root hair length, suggesting that maMYB functions in auxin-driven root hair growth. The maMYB RNAi mutants, however, still respond to auxin treatment and promote root hair growth, indicating that maMYB is not the sole regulator of auxin-dependent root hair growth (Slabaugh et al. 2011). How endoplasmic reticulum-localized maMYB functions in auxin signaling is not known and future studies should uncover further mechanistic details.

### Ethylene

Ethylene is another phytohormone that enhances root hair growth (Fig. 1). Different from auxin, ethylene can also induce ectopic root hairs from non-hair cell files. Interestingly, application of ethylene or its precursor, 1-aminocyclopropane-1-1carboxylic acid (ACC), induces *RSL4* expression (Fig. 3a) (Zhang et al. 2016). ETHYLENE INSENSITIVE 3 (EIN3) and its homolog EIN3-LIKE1 (EIL1) are key transcription factors to activate ethylene signaling. Both EIN3 and EIL1 are required for *RSL4* induction by ethylene (Feng et al. 2017). EIN3 binds to the EIN3-binding site within the *RSL4* promoter and directly regulates *RSL4* expression (Feng et al. 2017). Intriguingly, EIN3 physically interacts with RHD6 and they seem to promote *RSL4* expression synergistically in the presence of ethylene (Feng et al. 2017).

A C2H2 transcription factor ZINC FINGER PROTEIN 5 (ZFP5) also participates in the regulation of ethylene-dependent root hair growth. Intriguingly, the *zfp5-4* loss-of-function mutation blocks only root hair growth and not ectopic root hair formation after ACC treatment (An et al. 2012). Thus, ZFP5 seems to have a specific role to elongate root hairs in response to ethylene.

### Cytokinin

Cytokinin promotes root hair elongation and this regulation is independent from auxin or ethylene (Zhang et al. 2016). *ZFP5* expression is elevated by cytokinin and the *zfp5-4* mutant is insensitive to cytokinin for the hair growth phenotype (An et al. 2012), indicating that ZFP5 is an essential factor for the cytokinin-mediated root hair growth. ZFP5 directly activates *CPC* expression (An et al. 2012), thus ZFP5 appears to control root hair growth through *CPC*. Since ZFP5 does not affect root hair fate, it is also possible that ZFP5 regulates other factors that play more specific roles in hair growth.

### Abscisic acid

ABA has a negative effect on root hair growth (Schnall and Quatrano 1992) (Fig. 1). ABA induces expression of *OBP4*, which subsequently represses root hair growth (Rymen et al. 2017). Moreover, OBP4 directly suppresses *RSL2* expression. Thus, OBP4 inhibits root hair growth through *RSL2* in response to ABA (Rymen et al. 2017).

### Phosphate availability

Pi availability strongly influences root hair growth (Fig. 2). Regarding transcriptional regulations of Pi starvation, two MYB transcription factors, PHOSPHATE STARVATION RESPONSE 1 (PHR1) and its functionally redundant homolog PHR1-LIKE1 (PHL1) play a critical role on Pi starvation responses (Bustos et al. 2010; Rubio et al. 2001). Accordingly, the *phr1 phl1* double mutants are insensitive to Pi starvation since they do not show enhancement of root hair development under Pi limiting conditions (Bustos et al. 2010). The overexpression line of *PHR1* has much longer root hairs than those of wild type under Pi starvation condition (Bustos et al. 2010), indicating that PHR1 positively regulates root hair growth. Although *PHR1* expression does not strongly respond to Pi status (Rubio et al. 2001) and the activity of PHR1 is primarily regulated by SUMOylation (Miura et al. 2005) and inhibitory binding protein SPX1 and SPX2 (Puga et al. 2014), a recent study showed that ARF7 and ARF19 promote *PHR1* expression in roots (Huang et al. 2018). Thus, PHR1 may act downstream of auxin signaling. Auxin also acts systemically to transduce the Pi starvation signal since a recently proposed model suggests that Pi deficiency in soil is recognized at a root tip where auxin synthesis is enhanced by upregulation of TAA1 (Bhosale et al. 2018). Newly synthesized auxin is then transferred from the root tip to root hair cells by an auxin influx transporter AUXIN RESISTANT 1 (AUX1). After root hair cells uptake auxin, they induce cell elongation through transcriptional and non-transcriptional manners (Bhosale et al. 2018; Dindas et al. 2018).

Pi deficiency, in addition, affects fate of root hair cells and most likely ethylene, induced by Pi starvation, causes ectopic root hair formation. It is shown that Pi starvation increases the levels of the EIN3 protein which can promote root hair growth by direct transcriptional enhancement of root hair specific genes (Song et al. 2016). In addition, ALFIN-LIKE 6 (AL6) would be a key regulator of root hair formation for Pi starvation response. A homeodomain protein AL6 was isolated by forward genetic screening based on defects in root hair elongation under Pi starvation condition. AL6 induces root hair growth through activating *ETC1* expression (Chandrika et al. 2013). Although cell fate of root hairs has not been evaluated in detail, AL6 can regulate cell fate of root hairs though *ETC1*. Another potential regulator is *WRKY75* which is upregulated in response to Pi starvation (Devaiah et al. 2007). Surprisingly, however, WRKY75 is a negative regulator of root hair fate since it directly suppresses *CPC* expression (Devaiah et al. 2007). Thus, WRKY75 likely prevents root hair formation under low phosphate conditions. Although this seems paradoxical, having a negative regulator might be required for a precise response to rapidly changing environmental conditions.

How plants transduce high Pi stress is less understood but bHLH32 suppresses root hair development to reduce Pi uptake (Chen et al. 2007). Accordingly, the *bhlh32* mutant does not inhibit root hair growth under high Pi condition and this phenotype is accompanied by up-regulation of several Pi starvation-induced genes (Chen et al. 2007).

While Pi response has mostly been studied using high or low Pi medium, a new study was conducted using a micro-device, called Dual-flow-RootChip, that allows flow of different liquids on two sides of roots (Stanley et al. 2018). When an Arabidopsis root containing RSL4-GFP was exposed to high and low Pi medium, surprisingly the RSL4-GFP signal was enhanced on the high Pi side, resulting in the production of longer root hairs only on the high Pi side (Stanley et al. 2018). This striking observation demonstrates that plants’ response to local Pi signals is different from their systemic response and plants induce root hair growth when they face high Pi locally to promote efficient Pi uptake.

## Conclusions and perspectives

In this review we highlighted the key transcriptional regulations that link developmental and/or environmental inputs to root hair identity and growth. Various exogenous cues affect root hair growth and it is becoming increasingly evident that many of these upstream cues are integrated to auxin- or ethylene-mediated signaling pathways. Central regulators acting downstream of these signaling cascades are the two bHLH transcription factors RSL2 and RSL4 that function as a hub to regulate root hair growth in response to environmental signals. For cell fate specification, two MYB transcription factors CPC and ETC1 appear to function as a hub to integrate environmental and developmental cues. Another emerging characteristic of the root hair gene regulatory network is the intimate transcriptional interaction between these positive root hair regulators and negative regulators such as GTL1 and OBP4. In particular, GTL1 and RSL4 form a mutually regulating transcriptional module, and we predict that GTL1 functions as a stabilizer in this module to minimize the fluctuation of *RSL4* expression under the changing environment. Although many core regulators of root hair development have been identified (Table 1), many of their transcriptional interactions remain unknown. Further elucidation of these interactions will reveal the gene regulatory network of root hair development and should facilitate better understanding of how plants regulate cell fate and growth in response to environmental signals.

**Table 1:** Transcription factors involved in root hair development

Gene name	AGI	Type	Function	References
*CAPRICE (CPC)*	At2g46410	MYB	Cell fate	Wada et al. (1997)
*TRIPTYCHON (TRY)*	At5g53200	MYB	Cell fate	Schellmann et al. (2002)
*ENHANCER OF TRY AND CPC (ETC1)*	At1g01380	MYB	Cell fate	Simon et al. (2007)
*MYB23 (MYB23)*	At5g40330	MYB	Cell fate	Kang et al. (2009)
*WEREWOLF (WER)*	At5g14750	MYB	Cell fate	Lee and Schiefelbein (1999)
*ENHANCER OF GLABRA3 (EGL3)*	At1g63650	bHLH	Cell fate	Bernhardt et al. (2003)
*GLABRA3 (GL3)*	At5g41315	bHLH	Cell fate	Bernhardt et al. (2003)
*TRANSPARENT TESTA GLABRA2 (TTG2)*	At2g37260	WRKY	Cell fate	Johnson et al. (2002), Ishida et al. (2007)
*GLABRA (GL2)*	At1g79840	HD-ZIP	Cell fate	Masucci et al. (1996), Di Cristina et al. (1996), Lin et al. (2015)
*ZINC FINGER PROTEIN 5 (ZFP5)*	At1g10480	C2H2	Hormone response	An et al. (2012)
ETHYLENE INSENSITIVE 3 (EIN3)	At3g20770	EIL	Hormone response	Song et al. (2016), Feng et al. (2017)
EIN3-LIKE1 (EIL1)	At2g27050	EIL	Hormone response	Song et al. (2016), Feng et al. (2017)
AUXIN REPONSE FACTORs (ARFs)	–	ARF	Hormone response	Mangano et al. (2017), Bhosale et al. (2018), Choi et al. (2018)
*PHOSPHATE STARVATION RESPONSE 1 (PHR1)*	At4g28610	MYB	Phosphate response	Bustos et al. (2010)
*PHR1-LIKE1 (PHL1)*	At5g29000	MYB	Phosphate response	Bustos et al. (2010)
*ALFIN-LIKE6 (AL6)*	At2g02470	Homeodomain protein	Phosphate response	Chandria et al. (2013)
*WRKY75*	At5g13080	WRKY	Phosphate response	Devaiah et al. (2007), Rishmawi et al. (2014)
*BASIC LOOP HELIX LOOP 32 (BHLH32)*	At3g25710	bHLH	Phosphate response	Chen et al. (2007)
*ROOT HAIR DEFECTIVE6 (RHD6)*	At1g66470	bHLH	Hair growth	Masucci and Schiefelbein (1994), Menand et al. (2007)
*RHD SIX-LIKE 1 (RSL1)*	At5g37800	bHLH	Hair growth	Menand et al. (2007), Yi et al. (2010)
*RHD SIX-LIKE 2 (RSL2)*	At4g33880	bHLH	Hair growth	Yi et al. (2010), Rymen et al. (2017)
*RHD SIX-LIKE 3 (RSL3)*	At2g14760	bHLH	Hair growth	Bruex et al. (2012), Pires et al. (2013)
*RHD SIX-LIKE 4 (RSL4)*	At1g27740	bHLH	Hair growth	Yi et al. (2010) and etc
*RHD SIX-LIKE 5 (RSL5)*	At5g43175	bHLH	Hair growth	Pires et al. (2013)
*Lotus japonicus ROOT HAIR LESS-LIKE 1 (LRL1)*	At2g24260	bHLH	Cell fate & Hair growth	Karas et al. (2009), Lin et al. (2015), Breuninger et al. (2016)
*Lotus japonicus ROOT HAIR LESS-LIKE 2 (LRL2)*	At4g30980	bHLH	Cell fate & Hair growth	Karas et al. (2009), Lin et al. (2015), Breuninger et al. (2016)
*Lotus japonicus ROOT HAIR LESS-LIKE 3 (LRL3)*	At5g58010	bHLH	Hair growth	Karas et al. (2009), Lin et al. (2015), Breuninger et al. (2016)
*Lotus japonicus ROOT HAIR LESS-LIKE 4 (LRL4)*	At1g03040	bHLH	Hair growth	Bruex et al. (2012), Breuninger et al. (2016)
*Lotus japonicus ROOT HAIR LESS-LIKE 5 (LRL5)*	At4g02590	bHLH	Hair growth	Bruex et al. (2012), Breuninger et al. (2016)
*MEMBRANE ANCHORED MYB (maMYB)*	At5g45420	MYB	Hair growth	Slabaugh et al. (2011)
*HOMEODOMAIN GLABROUS 11 (HDG11)*	At1g73360	HD-ZIP	Hair growth	Xu et al. (2014)
*OBF BINDING PROTEIN 4 (OBP4)*	At5g60850	Dof	Hair growth	Rymen et al. (2017)
*GT2-LIKE1 (GTL1)*	At1g33240	Trihelix	Hair growth	Shibata et al. (2018)
*DF1*	At1g76880	Trihelix	Hair growth	Shibata et al. (2018)
